# Effect of radiation dose to the periventricular zone and subventricular zone on survival in anaplastic gliomas

**DOI:** 10.3332/ecancer.2019.956

**Published:** 2019-07-31

**Authors:** Deepthi Valiyaveettil, Monica Malik, Deepa M Joseph

**Affiliations:** 1 Department of Radiation Oncology, Nizam’s Institute of Medical Sciences, Punjagutta, Hyderabad 500082, India; 2 Department of Radiation Oncology, All India Institute of Medical Sciences, Rishikesh 249203, India

**Keywords:** subventricular zone, anaplastic gliomas, neurocognition, periventricular zone

## Abstract

**Purpose:**

Evidence suggests a correlation of subventricular zone (SVZ) irradiation on survival. Most of the data have been analysed in glioblastoma patients. The aim of this study is to analyse the dose to the subventricular and periventricular zone and its outcomes in anaplastic gliomas.

**Materials and methods:**

A retrospective analysis of patients with anaplastic gliomas were admitted for post-chemoradiation from January 2010 to June 2016 was done from treatment records. SVZ was contoured as 5 mm expansion along the lateral margin of the lateral ventricles, and PVZ was contoured as 5 mm lateral expansion adjacent to ventricles. Dosimetric data were collected from the treatment planning system.

**Results:**

Ninety-five patients were included in the analysis. The median age was 35 years. Two- and five-year overall survival (OS) for the entire group was 84% and 54.2%, respectively. Two- and five-year progression-free survival (PFS) was 79.8% and 50.6%, respectively. Patients receiving <54 Gy to the i/l SVZ showed a significantly better PFS and OS. 5-Year OS was 72.6% in this group compared to 37% for the group receiving ≥54 Gy (*p* = 0.01). Five-year PFS was 69.9% in this group compared to 31.9% for the group receiving ≥54 Gy (p = 0.02). However, this was not significant in multivariate analysis.

**Conclusion:**

Increased dose to the ipsilateral SVZ does not correlate with improved survival in anaplastic gliomas. There is conflicting evidence regarding the benefit of irradiating the stem cell zones. Future studies should focus on optimizing doses to these areas to reduce detriment in neurocognition.

## Background

High-grade gliomas (HGG) are the most common brain tumours. They are usually aggressive and difficult to control despite advances in multimodality management. Radiotherapy plays a critical role in the management of these tumours.

The origin of HGGs is still not clearly understood. Initially, they were hypothesized to originate from neoplastic transformation of glial cells [1, 2]. Later, the concept of cancer stem cells emerged [3, 4]. Recent research in glioblastoma suggests that the heterogeneity seen may be related to the cells of origin, which have stem-cell-like characteristics [3, 5, 6].

In an adult mammalian brain, a 3–5-mm-thick lateral periventricular region of the lateral ventricles [subventricular zone (SVZ)] and a subsection of the hippocampal formation (subgranular layer) are considered as germinal areas. They harbour normal neural stem cells that maintain the ability to generate neurons and glia throughout adulthood [7–9]. Periventricular zone (PVZ) is the region surrounding the ventricles. The SVZ has been described as the ‘largest niche of neurogenesis in the adult mammalian brain’ [8, 10, 11]. The neural stem cells (NSCs) in these areas are being studied extensively for therapeutic applications because of their ability to proliferate. These newly generated cells are able to migrate and differentiate into any of the main cell types of the central nervous system (CNS) [8, 9, 12, 13].

These cells also play an important role in the repair of injury within the CNS [14, 15], including recovery from radiation-induced damage [16, 17]. Stem cells within the SVZ and subgranular zone (SGZ) are thought to play functional roles in memory and neurocognition [7, 14, 18]. Damage to the NSCs during radiation therapy can have detrimental effects [19, 20].

A few retrospective studies demonstrated that patient survival and recurrence patterns in glioblastoma may be related to neuronal stem cells, located in the SVZ [5, 21]. Several groups have correlated higher radiation doses to the SVZ and SGZ areas with improved survival outcomes in glioblastoma patients [22–25]. Given this potential relationship between radiation doses to regions containing neural stem cells and survival outcomes in glioblastoma, we aimed to explore this further in anaplastic gliomas.

To test our hypothesis, we performed a retrospective analysis of radiation treatment plans of anaplastic glioma patients admitted for adjuvant radiation to study the effect of the radiation dose inadvertently delivered to the SVZ on the survival outcomes.

## Materials and methods

Patients with histopathologically diagnosed anaplastic gliomas consecutively treated in our institute from January 2010 to June 2016 were included in this retrospective analysis. Patients who failed to complete the radiation prescription were excluded from the study. Patient data, including demographics, imaging data, treatment and clinical outcomes were collected from treatment and follow-up records. Patient demographics, tumour characteristics and treatment details were correlated with survival outcomes.

### Treatment

All patients had undergone maximal safe resection. The extent of resection was identified as gross total or subtotal resection or biopsy by operative and clinical notes and when available by post-operative CT or MRI imaging. The planning target volume (PTV) for each patient was designated by the treating physician at the time of initial treatment planning.

The CT simulation scan was obtained with 3-mm slice thickness and was fused to pre- and postoperative MRI data. Gross tumour volume (GTV) included the resection cavity and any gross residual tumour observed on postoperative MRI. The clinical target volume (CTV) was defined by GTV and hyperintense area on T2 FLAIR MRI plus a 1.5–2-cm margin accounting for a potential microscopic extension. The PTV1 was CTV plus a 5-mm margin in all directions to account for daily setup uncertainty. A subsequent boost was given to PTV2, which was defined as GTV plus a 1-cm margin. Radiotherapy was delivered by three-dimensional conformal radiotherapy or intensity modulated radiotherapy (IMRT), with a standard dose of 60 Gy in 30 daily fractions, 5 days per week. Temozolomide was given as concurrent (75 mg/m^2^/day) and adjuvant chemotherapy (150–200 mg/m2 for 5 days every 28 days for 6–12 cycles).

### SVZ

Tumours were divided into groups (contacting and non-contacting) based on their spatial relationship to the SVZ. SVZs were contoured according to published guidelines [26] onaxial planning CT scans as 5-mm expansion along the lateral margin of the lateral ventricles. PVZ was contoured as 5-mm lateral expansion adjacent to ventricles. Tumour epicentre was used to assign laterality of the SVZ. Ipsilateral (i/l), contralateral (c/l) and bilateral (b/l) SVZ and PVZs were contoured on the co-registered MRI and CT images. Tumours were termed as SVZ contacting tumours if there was a distance of 0 mm between the contrast-enhancing tumour edge and the SVZ.

### Dosimetry

Treatment planning system was Eclipse version 8.6 (Varian Medical Systems). Dose-volume histograms were generated on the original plans and dose-volume parameters for each SVZ and PVZ volume were extracted. Volume and dose to the i/l, c/l, and b/l SVZ and PVZ were documented as a continuous variable.

### Statistics

Survival analysis: The study used progression-free survival (PFS) and overall survival (OS) as the main outcome determinants. PFS was calculated from the date of the initial surgical resection to disease progression, or to the last recorded date of follow-up without progression. OS was defined as the duration from the date of the primary surgery to the date of death or last contact. Survival interval was represented in months.

Overall survival was estimated as median and with 95% confidence interval (CIs). Kaplan–Meier (KM) survival curves were used to visualise the impact on survival for a significant prognostic factor. Log-rank test was used to estimate univariable significance for KM curves. Multivariate analysis was assessed using the Cox proportional hazards model, including significant covariates in univariate analysis. All reported *p*-values were two-sided. For all statistical tests, differences were considered significant at the 5% level. Statistical analysis was done using SPSS v20 software (IBM Corp. in Armonk, NY).

## Results

A total of 120 consecutive patient records were retrieved for this study. Ninety-five patients who had completed the course of radiation prescribed and had follow-up details were included in the study. The median age was 35 years, range 6–68 years. All patient characteristics are tabulated in Table 1.

The most common symptom at the time of presentation was headache (57.8%), followed by seizures, nausea and vomiting and hemiparesis. 55 patients (58%) had tumours confined to single lobe, frontal lobe being the most common. 72 patients (75.7%) had tumours contacting the subventricular region radiologically.

All the patients underwent maximal safe resection. Gross total excision was done in 27.3%. 51.5% were anaplastic astrocytomas and 32.6% had anaplastic oligodendrogliomas as histopathology. Immunohistochemistry (IHC) testing was available in our institute from 2014. IHC data including IDH1 mutational analysis, ATRX, ki-67 and 1p19q co-deletion testing were available in 37.8% of cases.

The majority of patients (84.2%) had performance status (PS) of ≤2 by ECOG scoring system at the time of presentation during RT. 84 (88.4%) received concurrent chemotherapy with temozolomide and 72 (75.7%) patients received adjuvant chemotherapy. Median number of adjuvant chemotherapy cycles was six.

Median doses received by i/l, c/l and b/l SVZ were 54 Gy, 37 Gy and 45 Gy, respectively. The median dose received by i/l, c/l and b/l PVZ was 53 Gy, 37 Gy and 45 Gy, respectively. All dosimetric data are tabulated in Table 2.

### Survival analysis

To reduce the heterogeneity of the population, survival analysis excluded children and tumours involving cerebellum. 85 patients were included. Median follow up was 39 months (Range: 5–92 months).

2-year and 5-year OS for the entire group was 87.5% and 55.7%, respectively. 2-year and 5-year PFS for the entire group was 83.3% and 52.3%, respectively. Age and gender did not significantly impact OS or PFS.

**Figure 1.**
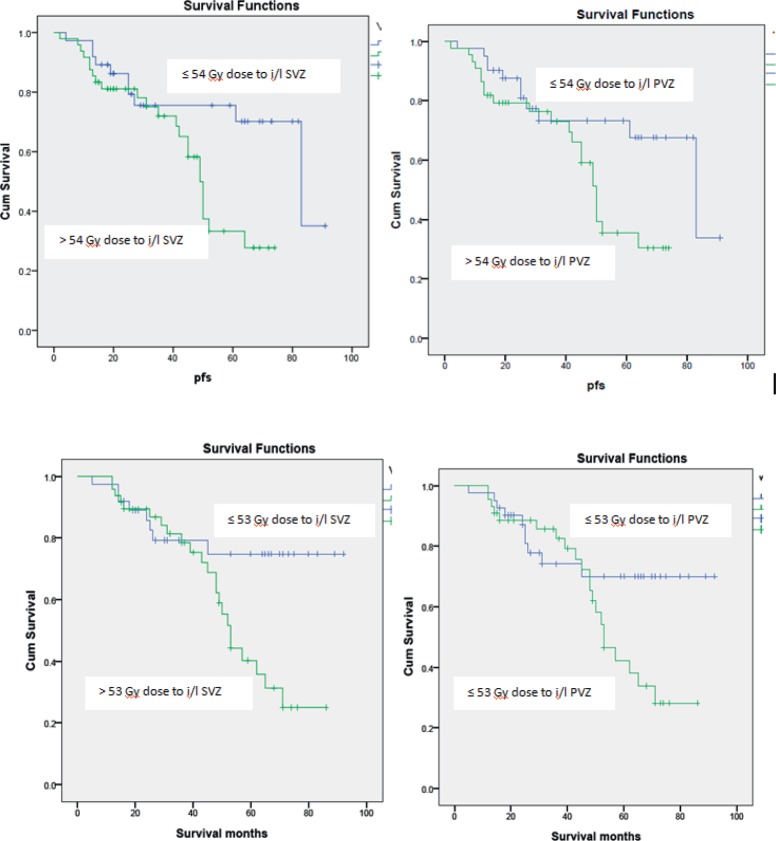
Kaplan–Meier estimates for progression free survival and overall survival.

Patients receiving ≤54 Gy to the i/l subventricular zone showed a significantly better PFS. 2- and 5-year PFS was 86.2% and 75.5% in this group compared to 81.1% and 33.3% for the group receiving > 54 Gy (*p* = 0.01), respectively. 2- and 5-year PFS was 87.5% and 79.3% in the group receiving <52 Gy to the i/l periventricular zone compared to 69.3% and 41.2% for group receiving ≥ 52 Gy (*p* = 0.01), respectively. However, the multivariate analysis did not show significant results. Other dosimetric correlations are documented in Table 3 and Kaplan–Meier survival estimates are shown in Figure 1.

During follow-up, 22 (23.1%) patients developed progression, of which 18 patients received salvage treatment with surgery in 15 patients and re-irradiation in six patients. Three patients received concurrent and adjuvant temozolomide during re-irradiation.

## Discussion

According to the cancer stem cells (CSC) hypothesis, all CSCs need to be eliminated in order to cure cancer [27]. The SVZ and PVZ are hypothesized as germinal centres which house these cells. Tumour contact with these regions has shown to be an independent adverse prognostic factor in many studies [28].

In a retrospective analysis, 53 patients with newly diagnosed glioblastoma were classified based on their spatial relationship to the SVZ. Results showed that patients whose tumours made contact with the SVZ had a significantly higher incidence of multifocal disease at diagnosis and also had decreased OS and PFS [5]. A similar analysis of 91 glioblastoma patients showed significantly reduced PFS in patients whose tumour had contact with both the SVZ and the cortex [21]. In our study, tumours contacting the SVZ preoperatively had reduced survival compared to the non-contacting group but this result was not statistically significant.

Preclinical studies and retrospective clinical evidence [22–25, 29–31] have established a correlation between radiation dose to the SVZ and survival, suggesting a favourable impact of irradiation to the SVZ. Most of these studies included only glioblastomas.

The first retrospective study [25] to analyse the effect of a high dose of radiation to neural stem cell niches included 55 adult patients with Grade 3 or Grade 4 gliomas. This study showed significant improvement in PFS in patients whose b/l SVZ received a dose of 43 Gyor more (15.0 vs 7.2 months; *p* = 0.028). There was no improvement in overall survival. This study included 17 (30%) patients with anaplastic gliomas.

A retrospective pooled analysis of glioblastoma patients by Lee *et al* [23] showed that patients receiving more than 59.4 Gy to the i/l SVZ reported improvements in both PFS and OS. Another retrospective analysis conducted in India by Gupta *et al* [24] observed improvement in overall survival in glioblastoma patients treated with higher mean dose to the i/l SVZ.

A recent retrospective study [31] was conducted to identify SVZ related prognostic factors for survival and recurrence patterns. This study included 43 patients with primary glioblastoma. Results showed contact to SVZ, as well as reduced b/l SVZ radiation dose coverage (V20Gy <84%), might be independent poor prognostic factors for time to progression (7 months versus 5.2 months, *p* = 0.017) on multivariate analysis.

These studies suggest that higher doses targeted to the SVZ improve the progression-free survival [25] (PFS) of patients compared to dose targeted to the tumour alone. Additionally, increasing the mean radiation dose to the i/l SVZ was associated with significantly improved OS [22, 24]. These results are important and suggest that higher radiation dose to the SVZ may be beneficial in terms of survival outcomes.

In tune with these reports, a prospective study of hypofractionated radiation therapy found improved survival in long-term survivors with necrosis in the SVZ [32]. A recent single arm clinical trial investigating planned neural stem cell niche irradiation in glioblastoma [33] showed mean dose of 58 Gy or greater to the i/l SVZ correlated positively with improved overall survival (16 months versus 14 months, *p* = 0.03).

All these studies point towards increasing the dose of radiation for improved survival. On the contrary, there have been studies which showed detrimental results with increased SVZ doses. A study by Elicin *et al* [30] found a worse PFS among patients with high i/l SVZ dose (>62.25 Gy) in both subgroups of good performance status and SVZ without tumoural contact. A worse PFS was also found among patients with higher doses to c/l SVZ >59.2 Gy (7.1 versus 10.4 months), but it was not confirmed in multivariate analysis.

A recent prospective study [34] conducted showed patients who received <59 Gy to the i/l SVZ had significantly better outcomes than patients who received greater than 59 Gy to the i/l SVZ both in terms of median PFS (20.5 versus 9.7 months, *p* = 0.016) and OS (20.6 versus 13.3 months, *p* = 0.026). Similar to these results, our study has also showed that patients receiving <54 Gy to the i/l SVZ had significantly better PFS and OS at 5 years, although our study included only patients with anaplastic glioma histology.

Although these studies show that the plasticity of the SVZ has quantifiable benefits for the adult brain, it comes with a potential limitation. Studies in patients with gliomas have suggested a potential risk of developing neurocognitive sequelae when the NSCs are irradiated during radiotherapy, most notably in children [35–40]. Although an association between the RT dose to the hippocampus and neurocognitive function has been studied, the relationship between RT dose to the SVZ and neurocognitive function is controversial [41–43].

The available literature on the effects of irradiation of SVZ in anaplastic gliomas is sparse. The prognosis of anaplastic gliomas is better compared to glioblastoma. Many of these patients have reported longer survival. Therefore, the implications of neurocognitive effects should be kept in mind. Irradiation of SVZ in these patients remains an interesting academic question which is likely to have important implications for radiation treatment planning strategies in the future. The present evidence of subventricular zone dose in glioblastoma shows contradictory results. Our study could not demonstrate any benefit from higher doses to SVZ or PVZ in anaplastic gliomas.

Limitations: Our study has inherent biases of a retrospective study. Immuno-histochemistry was not available in all patients. Neuro-cognition pre and post RT was not tested. The patterns of failures were not analysed in relation to any of the SVZ dose-volume parameters.

## Conclusions

Increased dose to the ipsilateral subventricular zone does not correlate with improved survival in anaplastic gliomas. There is conflicting evidence in the literature regarding the benefit of irradiating the stem cell zones in gliomas. This may suggest that the relationship between these zones and survival is not clearly understood. Future studies should focus on optimising doses to these areas to reduce detriment in neurocognition.

## Conflicts of interest

All authors declare no conflict of interest

## Authors’ contributions

All authors contributed substantially to this work.

## Funding/financial support received

The authors received no funding for this article.

## Figures and Tables

**Table 1.** Patient, disease and treatment characteristics.

Patient variables (*N* = 65)	No. of patients (%)
Mean and median age in years (range)	35 and 35.29 (6–68)
Sex Male Female	62 (65.3%) 33 (34.7%)
ECOG performance status ≤2 >2	80 (84.2%) 15 (15.7%)
Chief presenting complaints Headache Seizures Nausea & vomiting Weakness and paraesthesias	55 (57.8%) 44 (49.3%) 20 (21.0%) 18 (18.9%)
Mean duration of symptoms in months	8.2 months
Location of tumour Confined to single lobe Two lobes Three lobes Cerebellum Brainstem	55 (57.98%) 34-frontal (61.8%) 28 (29.4%) 1 (1.05%) 5 (5.2%) 3 (3.1%)
Tumour contacting SVZ Yes No	N (%) 72 (75.7%) 23 (24.2%)
Extent of resection Gross total resection Subtotal resection Biopsy/decompression	26 (27.3%) 59 (62.1%) 10 (10.5%)
Post op histopathology Anaplastic astrocytoma (AA) Anaplastic oligodendroglioma Anaplastic oligoastrocytoma Anaplastic pilocytic astrocytoma AA with gemistocytic variant Diffuse fibrillary astrocytoma with anaplasia	49 (51.5%) 31 (32.6%) 8 (8.4%) 5 (5.2%) 1 (1.05%) 1 (1.05%)
Immunohistochemistry Yes No	36 (37.8%) 59 (62.1%)
Radiotherapy technique 3DCRT IMRT	17 (17.8%) 78 (82.1%)
Concurrent chemotherapy with TMZ Yes No	84 (88.4%) 11 (11.5%)
Adjuvant chemotherapy with TMZ Yes No Mean number of cycles **>**6 cycles	72 (75.7%) 23 (24.2%) 6 16 (22.2%)

*N* = Total number of patients in the study, ECOG = Eastern Cooperative Oncology Group, SVZ = Subventricular zone, 3DCRT = Three-dimensional conformal radiotherapy, IMRT = Intensity-modulated radiotherapy, TMZ = Temozolomide

**Table 2.** Dosimetric data.

Dosimetric variables	Dose in gray
**Volumes** Ipsilateral SVZ Contralateral SVZ Bilateral SVZ Ipsilateral PVZ Contralateral PVZ Bilateral PVZ	**Mean ± SD** 6.41 ± 5.6 7.40 ± 3.56 12.86 ± 5.05 13.80 ± 6.22 17.52 ± 6.39 30.99 ± 11.62
**Doses** Ipsilateral SVZ Contralateral SVZ Bilateral SVZ Ipsilateral PVZ Contralateral PVZ Bilateral PVZ	**Mean ± SD, Median** 51.73 ± 8.91, 54 36.79 ± 8.75, 37 42.82 ± 8.73, 45 50.02 ± 10.24, 53 36.33 ± 8.70, 37 42.60 ± 8.54, 45

SVZ = subventricular zone, PVZ = periventricular zone, SD = Standard Deviation

**Table 3.** Survival analysis and dosimetric correlations.

Variable (*n*)	2 year OS in %	5 year OS in %	*p* value	2 year PFS in %	5 year PFS in %	*p* value
Age ≤35 years (*n* = 46) >35 years (*n* = 39)	89.1 70.2	61.8 49.4	0.54	84.8 81.4	48.7 55.3	0.47
Sex Male (*n* = 56) Female (*n* = 29)	78.3 93.0	54.2 59.5	0.55	81.8 86.2	49.1 58.2	0.31
SVZ contacting Yes (*n* = 69) No (*n* = 16)	85 100	53.8 63.5	0.27	80.9 93.8	49.9 62.5	0.30
SVZ i/l < 54 Gy (*n* = 37) ≥ 54 Gy (*n* = 48)	85.8 89.4	74.8 40.2	**0.01**	86.2 81.1	75.5 33.3	**0.01**
SVZ c/l < 37 Gy (*n* = 41) ≥ 37 Gy (*n* = 44)	85.1 93.1	58.1 53.5	0.76	82.6 84.0	54.6 50.3	0.75
SVZ b/l < 44 Gy (*n* = 42) ≥ 44 Gy (*n* = 43)	77.2 97.7	59.2 51.6	0.98	75.1 90.9	60.5 45.5	0.94
PVZ i/l < 52 Gy (*n* = 41) ≥ 52 Gy (*n* = 44)	87.2 88.5	70.2 42.3	0.06	87.5 79.3	73.3 35.4	**0.03**
PVZ c/l < 37 Gy (*n* = 42) ≥ 37 Gy (*n* = 43)	80.0 95.3	60.6 52.0	0.69	80.6 86.0	61.2 45.2	0.46
PVZ b/l < 44 Gy (*n* = 42) ≥ 44 Gy (*n* = 43)	76.2 97.8	65.5 47.8	0.64	76.5 89.1	69.3 41.2	0.19

SVZ = subventricular zone, PVZ = periventricular zone, i/l = ipsilateral, c/l = contralateral, b/l = bilateral
